# Beliefs and attitudes about addressing alcohol consumption in health care: a population survey in England

**DOI:** 10.1186/s12889-018-5275-2

**Published:** 2018-03-21

**Authors:** Amy O’Donnell, Latifa Abidi, Jamie Brown, Nadine Karlsson, Per Nilsen, Kerstin Roback, Janna Skagerström, Kristin Thomas

**Affiliations:** 1 Institute of Health and Society, Newcastle University, Newcastle upon Tyne, NE2 4AX UK; 2 Department of Health Promotion, Maastricht University, Maastricht, Limburg Netherlands; 3 Research Department of Behavioural Science and Health, University College London, London, UK; 4 Research Department of Clinical, Educational and Health Psychology, University College London, London, UK; 5 Department of Medical and Health Sciences, Faculty of Medicine and Health, Linköping University, Linköping, Sweden; 6 Research and Development Unit, and Department of Medical and Health Sciences, Linköping University, Linköping, Sweden

**Keywords:** Brief intervention, Alcohol drinking, Prevention, Alcohol toolkit study, Population-based, Implementation

## Abstract

**Background:**

Despite robust evidence for their effectiveness, it has proven difficult to translate alcohol prevention activities into routine health care practice. Previous research has identified numerous provider-level barriers affecting implementation, but these have been less extensively investigated in the wider population. We sought to: (1) investigate patients’ beliefs and attitudes to being asked about alcohol consumption in health care; and (2) identify the characteristics of those who are supportive of addressing alcohol consumption in health care.

**Methods:**

Cross-sectional household interviews conducted as part of the national Alcohol Toolkit Study in England between March and April 2017. Data were collected on age, gender, social grade, drinking category, and beliefs and attitudes to being asked about alcohol in routine health care. Unadjusted and multivariate-adjusted logistic regression models were performed to investigate associations between socio-demographic characteristics and drinking category with being “pro-routine” (i.e. ‘agree completely’ that alcohol consumption should be routinely addressed in health care) or “pro-personal” (i.e. ‘agree completely’ that alcohol is a personal matter and not something health care providers should ask about).

**Results:**

Data were collected on 3499 participants, of whom 50% were “pro-routine” and 10% were “pro-personal”. Those in social grade C1, C2, D and E were significantly less likely than those in AB of being “pro-routine”. Women were less likely than men to be “pro-personal”, and those aged 35–44 or 65 years plus more likely to be “pro-personal” compared with participants aged 16–24. Respondents aged 65 plus were twice as likely as those aged 16–24 to agree completely that alcohol consumption is a personal matter and not something health care providers should ask about (OR 2.00, 95% CI 1.34–2.99).

**Conclusions:**

Most adults in England agree that health care providers should routinely ask about patients’ alcohol consumption. However, older adults and those in lower socio-economic groups are less supportive. Drinking status appears to have limited impact on whether people believe that alcohol is a personal matter and not something health care providers should ask about.

**Registration:**

Open Science Framework (https://osf.io/xn2st/).

## Background

Excessive alcohol consumption is a significant risk to public health [1], and the fifth leading global risk factor for morbidity and premature death [2]. As part of a wider strategy to address the adverse impacts of heavy drinking, screening and brief interventions provide a clinically- and cost-effective means of identifying and addressing alcohol-related problems, particularly when delivered in primary care settings [3–5].

However, it has proven difficult to translate this scientific knowledge into widespread implementation of alcohol prevention activities in routine health care [6–9]. In an effort to address this evidence to practice gap, England has seen the introduction of a range of policy measures to encourage the delivery of screening and brief alcohol interventions in primary care. These have included the development and dissemination of expert guidelines [10], and the application of targeted pay-for-performance schemes [11]. More recently, alcohol consumption questions have been incorporated within the National Health Service (NHS) Health Check for adults aged 40–75, and in April 2015, the national Enhanced Service for alcohol incentive scheme was replaced by a contractual requirement for practices to identify newly registered adult patients drinking above recommended levels [12]. However, whilst financial incentives seem to have some impact [13, 14], overall evidence suggests there has been limited progress towards the effective implementation of alcohol prevention in English primary care [15, 16].

Previous research has identified numerous individual-level barriers with regard to health care providers’ alcohol-preventive work, including: perceived lack of time; insufficient knowledge about early symptoms of problematic alcohol use; poor confidence in the ability to intervene with alcohol problems; resistance to raising the issue of alcohol with patients who are not seeking help for alcohol-related problems; and lack of awareness of safe drinking guidelines [17–20]. Further, many organizational barriers have been identified, including insufficient management and leadership support for alcohol-preventive efforts, lack of resources and financial incentives, and poor training resources [21–23].

Factors that affect implementation of alcohol prevention in health care have been less extensively investigated from the perspective of patients and the wider population. There has been some research into patient attitudes toward alcohol and other lifestyle risk factors being raised in health care consultations [24–26]. However, most of these studies have focused on the recall of and satisfaction with such conversations. There is evidence to suggest patients are generally favourable to discussing alcohol issues with health care providers [27, 28]. Yet these studies have been mostly conducted on a small, local scale, and focused on clinical populations. There is less knowledge about the beliefs and attitudes of the general public in relation to the delivery of alcohol prevention in everyday health care. A population-based survey conducted in Sweden in 2011 [29] showed that there was considerable support for routinely asking patients about their alcohol consumption in health care. However, the study found support was lower amongst excessive and hazardous drinkers, and those who were younger or less educated. No previous population-based study in England has investigated this issue, which has relevance for ongoing efforts to improve implementation of alcohol prevention in health care.

The aims of the current study were twofold. First, to investigate patients’ beliefs and attitudes to being asked about alcohol consumption in health care, and second, to identify the characteristics of those who are most and least supportive, respectively, of addressing alcohol consumption in health care.

## Methods

### Design and participants

The study employed a cross-sectional design using data from the Alcohol Toolkit Study (ATS). The ATS consists of household computer-assisted survey questionnaires carried out by Ipsos Mori of approximately 1700 adults per month. This study used two waves of data collected between March and April 2017, a total of 3499 participants. The ATS uses a type of random location sampling, which is a hybrid between random probability and simple quota sampling (see www.alcoholinengland.info or the published protocol [30] for more details). Participants included adults aged 16 and over, living in households in England.

### Measures

Data were collected on age, gender, and social grade. Social grade was measured using the British National Readership Survey (NRS) Social-Grade Classification Tool [31]. On the basis of details relating to the chief income earner in the household, interviewers classified participants into: AB: higher or intermediate managerial, administrative or professional; C1: supervisory or clerical and junior managerial administrative or professional; C2: skilled manual workers; D: Semi and unskilled manual workers; or E: Casual or lowest grade workers, pensioners and others who depend on the welfare state for their income.

Drinking of alcohol was assessed using the Alcohol Use Disorders Identification Test (AUDIT) [32]. AUDIT consists of 10 questions relating to: alcohol consumption (items 1–3); alcohol dependence (items 4–6) and alcohol-related harm (items 7–10). Based on their response, participants were dichotomised into two drinking categories: lower-risk drinkers (score of < 8) or risky drinkers (score of ≥8). The lower-risk drinking group also included abstainers.

Beliefs and attitudes to being asked about alcohol in routine health care were investigated using five items, with possible response options of “agree completely”, “agree to a large degree”, “agree to a small degree”, “do not agree”, and “don’t know” to each statement:

1. Health care providers (doctors, nurses, etc.) should routinely ask about patients’ alcohol consumption;
2. Health care providers should ask about patients’ alcohol consumption, but only if patients seek health care to discuss symptoms that could be related to high alcohol consumption;
3. Health care providers should ask about patients’ alcohol consumption, but only if the issue is brought up by the patient;
4. I believe people answer honestly when they are asked about their alcohol consumption at health care visits;
5. Alcohol consumption is a personal matter and not something health care providers should ask about.

### Analyses

Socio-demographic characteristics were analysed using descriptive statistics. Two-way tables of patient beliefs and attitudes about when alcohol should be addressed in health care versus drinking categories were performed and analysed using the chi-squared test. If the overall chi-square test was found to be statistically significant (*p* < .05), pairwise comparisons were conducted. For pairwise comparisons, Bonferroni correction for multiple comparisons was performed. Responses were dichotomised into agree completely and all other response categories.

Logistic regression models were performed to investigate associations between socio-demographic characteristics and drinking category with being “pro-routine” (i.e. expressing a belief that alcohol consumption should be routinely addressed in health care) or “pro-personal” (i.e. expressing a belief that alcohol is a personal matter and not something health care providers should ask about). In Model 1, the logistic regression analysis was unadjusted; in Model II, regression was multivariate-adjusted for age (categorical variable), sex, social grade, and risky drinking category. All statistical analyses were performed using SPSS 24. Results were considered significant at *p* < 0.05 using two-tailed tests. The plan was registered on the Open Science Framework prior to data analysis (https://osf.io/xn2st/).

### Ethical approval

The Smoking and Alcohol Toolkit Study is approved by the UCL Ethics Committee (ID 2808/005). In accordance with our ethical approval, all respondents were given a written information sheet about the study, and provided informed verbal consent.

## Results

### Sample characteristics

Data were collected on 3499 participants between March and April 2017, with 3484 providing complete data on all variables. Eighty-six percent of respondents were categorized as lower-risk (AUDIT score < 8) and 14% as risky drinkers (AUDIT score ≥ 8). Table 1 shows that compared to abstainers and lower-risk drinkers, risky drinkers were significantly more likely to be male (67.5% men vs 46.7% men), aged 16–24 years old (22.7% vs. 12.6%) and to have social grade C1 (39.5% vs 30.0%).

**Table 1:** Sociodemographic characteristics according to the two drinking categories

Variables	Total, *n* (%)	Low-risk drinking, *n* (%)	Risky drinking, *n* (%)
Sex	3484	3013	471
Men	1726 (49.5%)	1408 (46.7%)	318 (67.5%)
Women	1758 (50.5%)	1605 (53.3%)	153 (32.5%)
Age	3484	3013	471
16–24 years	488 (14.0%)	381 (12.6%)	107 (22.7%)
25–34 years	473 (13.6%)	405 (13.4%)	68 (14.4%)
35–44 years	503 (14.4%)	438 (14.5%)	65 (13.8%)
45–54 years	543 (15.6%)	457 (15.2%)	86 (18.3%)
55–64 years	527 (15.1%)	450 (14.9%)	77 (16.3%)
65 years or older	950 (27.3%)	882 (29.3%)	68 (14.4%)
Social grade	3484	3013	471
AB	907 (26.0%)	788 (26.2%)	119 (25.3%)
C1	1091 (31.3%)	905 (30.0%)	186 (39.5%)
C2	641 (18.4%)	553 (18.4%)	88 (18.7%)
D	512 (14.7%)	463 (15.4%)	49 (10.4%)
E	333 (9.6%)	304 (10.1%)	29 (6.2%)

Low-risk drinking = AUDIT summary score 0–7; Risky drinking = AUDIT summary score 8 or more

### Beliefs and attitudes about alcohol prevention in routine health care

Approximately 50% of respondents agreed completely, and 87.9% agreed completely or to a large extent, that health care providers should ask about patients’ alcohol consumption on a routine basis. Fewer respondents agreed completely that health care providers should only ask about patients’ alcohol consumption if patients seek health care to discuss symptoms that could be related to high consumption (29%), or only if the issue is brought up by the patient (21%). Ten percent agreed completely that alcohol consumption is a personal matter and not something that health care providers should ask about. Responses were cross-tabulated with drinking categories (lower-risk versus risky drinkers, see Table 2). Risky drinkers were less likely than lower-risk drinkers to believe that people answer honestly when they are asked about their alcohol consumption in routine health care visits. However there were no statistically significant differences by drinking category for all other questions.

**Table 2:** Beliefs and attitudes about alcohol prevention according to the two drinking categories

	Total, *n* (%)	Low-risk drinking, *n* (%)	Risky drinking, *n* (%)	*p*-value
Health care providers should routinely ask about patients’ alcohol consumption	3442	2973	469	0.052
Agree completely	1737 (50.5%)	1519 (51.1%)	218 (46.5%)	
Agree to a large or some extent	1287 (37.4%)	1088 (36.6%)	199 (42.4%)	
Do not agree	418 (12.1%)	366 (12.3%)	52 (11.1%)	
Alcohol consumption is a personal matter and not something health care providers should ask about	3430	2962	468	0.327
Agree completely	360 (10.5%)	320 (10.8%)	40 (8.5%)	
Agree to a large or some extent	809 (23.6%)	698 (23.6%)	111 (23.7%)	
Do not agree	2261 (65.9%)	1944 (65.6%)	317 (67.7%)	
Health care providers should ask about patients’ alcohol consumption, but only if patients seek health care to discuss symptoms that could be related to high consumption	3415	2947	468	0.744
Agree completely	977 (28.6%)	843 (28.6%)	134 (28.6%)	
Agree to a large or some extent	1201 (35.2%)	1043 (35.4%)	158 (33.8%)	
Do not agree	1237 (36.2%)	1061 (36.0%)	176 (37.6%)	
Health care providers should ask about patients’ alcohol consumption, but only if the issue is brought up by the patient	3423	2956	467	0.886
Agree completely	733 (21.4%)	637 (21.5%)	96 (20.6%)	
Agree to a large or some extent	1097 (32.0%)	945 (32.0%)	152 (32.5%)	
Do not agree	1593 (46.5%)	1374 (46.5%)	219 (46.9%)	
I believe people answer honestly when they are asked about their alcohol consumption at health care visits	3366	2902	464	0.007
Agree completely	331 (9.8%)	294 (10.1%)	37 (8.0%)	
Agree to a large or some extent	1173 (34.8%)	1034 (35.6%)	139 (30.0%)	
Do not agree	1862 (55.3%)	1574 (54.2%)	288 (62.1%)	

### Characteristics of participants with “pro-routine” beliefs and attitudes

Social grade (Table 3) was significantly associated with being “pro-routine” (i.e. those who agreed completely with the statement that health care providers should routinely ask about patients’ alcohol consumption). Findings from the crude model (Model I) were similar to those obtained in the multivariate model (Model II). In both models, those in all other social grades were less likely than those in AB of being “pro-routine” (Table 3), although this was not significant for social grade E in Model II. Drinking category was not statistically significantly associated in Model II with being “pro-routine”. Neither sex nor age were associated with being “pro-routine”.

**Table 3:** Odds ratios for being “pro-routine”: believing that health care providers should routinely ask about patients’ alcohol consumption

	Model I (Crude)^a^					Model II (Multivariate)^b^				
Variables	N	*n* (%)	OR	95% CI	p-value	N	*n* (%)	OR	95% CI	*p*-value
Sex										
Men	1717	850 (49.5%)	1			1707	848 (49.7%)	1		
Women	1738	890 (51.2%)	1.07	0.94–1.22	0.32	1735	889 (51.2%)	1.05	0.92–1.21	0.47
Age										
16–24 years	485	234 (48.2%)	1			484	234 (48.3%)	1		
25–34 years	466	249 (53.4%)	1.23	0.95–1.59	0.11	466	249 (53.4%)	1.19	0.92–1.54	0.18
35–44 years	498	241 (48.4%)	1.01	0.78–1.29	0.96	496	240 (48.4%)	0.92	0.71–1.18	0.50
45–54 years	540	273 (50.6%)	1.10	0.86–1.40	0.46	535	271 (50.7%)	1.03	0.80–1.32	0.82
55–64 years	525	265 (50.5%)	1.09	0.85–1.40	0.48	523	265 (50.7%)	1.02	0.79–1.31	0.91
65 years or older	941	478 (50.8%)	1.11	0.89–1.38	0.36	938	478 (51.0%)	1.01	0.81–1.27	0.92
Social grade										
AB	901	515 (57.2%)	1			898	514 (57.2%)	1		
C1	1087	536 (49.3%)	0.73	0.61–0.87	<.001	1082	536 (49.5%)	0.73	0.61–0.87	0.001
C2	636	293 (46.1%)	0.64	0.52–0.79	<.001	635	293 (46.1%)	0.63	0.51–0.78	<.001
D	506	232 (45.8%)	0.64	0.51–0.79	<.001	502	230 (45.8%)	0.62	0.50–0.77	<.001
E	325	164 (50.5%)	0.76	0.59–0.99	0.04	325	164 (50.5%)	0.83	0.68–1.02	0.08
Drinking excessively†										
AUDIT< 8	2973	1519 (51.1%)	1			2973	1519 (51.1%)	1		
AUDIT> = 8	469	218 (46.5%)	0.83	0.68–1.01	0.06	469	218 (46.5%)	0.83	0.68–1.02	0.08

†Risky drinking defined as: Low-risk drinking (AUDIT< 8); High-risk drinking (AUDIT > = 8)

OR, odds ratios; CI, confidence interval

^a^Model I is crude

^b^Model II is adjusted for sex, age, social grade and drinking status

### Characteristics of participants with “pro-personal” beliefs and attitudes

Women were less likely than men to be “pro-personal” in both Model I and Model II (Table 4). Both models indicated that those aged 35–44 or 65 years were more likely to be “pro-personal” compared with participants aged 16–24 years. Model II shows that social grades C2, D and E were associated with a higher likelihood than social grade AB of being “pro-personal” (Table 4). Drinking category was not statistically significantly associated in Model II with being “pro-personal”.

**Table 4:** Odds ratios for being “pro-personal”: viewing alcohol consumption as a personal matter and not something health care providers should ask patients about

	Model 1 (Crude)^a^					Model II (Multivariate)^b^				
Variables	N	*n* (%)	OR	95% CI	*p*-value	N	*n* (%)	OR	95% CI	*p*-value
Sex										
Men	1710	206 (12.0%)	1			1700	206 (12.1%)	1		
Women	1733	154 (8.9%)	0.71	0.57–0.89	0.003	1730	154 (8.9%)	0.68	0.54–0.85	0.001
Age										
16–24 years	482	35 (7.3%)	1			481	35 (7.3%)	1		
25–34 years	463	50 (10.8%)	1.55	0.98–2.43	0.06	463	50 (10.8%)	1.56	0.99–2.46	0.06
35–44 years	496	58 (11.7%)	1.69	1.08–2.63	0.02	494	58 (11.7%)	1.87	1.20–2.92	0.006
45–54 years	538	53 (9.9%)	1.40	0.89–2.18	0.14	533	53 (9.9%)	1.47	0.94–2.31	0.09
55–64 years	528	42 (8.0%)	1.10	0.69–1.76	0.68	526	42 (8.0%)	1.15	0.72–1.85	0.56
65 years or older	936	122 (13.0%)	1.91	1.29–2.84	0.001	933	122 (13.1%)	2.00	1.34–2.99	0.001
Social grade										
AB	905	70 (7.7%)	1			902	70 (7.8%)	1		
C1	1083	97 (9.0%)	1.17	0.85–1.62	0.33	1078	97 (9.0%)	1.28	0.92–1.77	0.14
C2	633	89 (14.1%)	1.95	1.40–2.72	<.001	632	89 (14.1%)	2.05	1.47–2.87	<.001
D	499	63 (12.6%)	1.72	1.20–2.47	0.003	495	63 (12.7%)	1.85	1.29–2.67	0.001
E	323	41 (12.7%)	1.73	1.15–2.61	0.008	323	41 (12.7%)	1.82	1.20–2.75	0.005
Drinking excessively†										
AUDIT< 8	2962	320 (10.8%)	1			2962	320 (10.8%)	1		
AUDIT > = 8	468	40 (8.5%)	0.77	0.55–1.09	0.14	468	40 (8.5%)	0.80	0.56–1.14	0.21

†Risky drinking defined as: Low-risk drinking (AUDIT< 8); High-risk drinking (AUDIT > = 8)

OR, odds ratios; CI, confidence interval

^a^Model I is crude

^b^Model II is adjusted for sex, age, social grade and drinking status

### Sensitivity analyses

Sensitivity analyses of AUDIT as a continuous explanatory variable were conducted in order to assess whether the lack of statistical significance was due to the dichotomization of AUDIT (lower-risk drinking vs. risky drinking) and a subsequent loss of statistical power. Using the Box–Tidwell approach, the linearity of the relationship between AUDIT and its log transformation on the outcomes “pro-routine” and “pro-personal” was analysed. For the outcome “pro-routine”, the interaction of AUDIT and its log transformation was not statistically significant (*p* = 0.95), indicating no evidence of a non-linear relationship. However, when AUDIT was analysed as a continuous predictor in the logistic regression model, results showed that AUDIT is not statistically significantly associated with pro-routine (OR = 0.98 95% CI (0.97, 1.00); *p* = 0.052). For the outcome “pro-personal”, the interaction of AUDIT and its log transformation is statistically significant (*p* = 0.004) indicating evidence of a non-linear relationship. A Box Tidwell transformation was conducted to linearize AUDIT in order to analyse AUDIT as a continuous predictor. Logistic regression analyses show that the odds ratio of pro-personal is not associated with AUDIT (*p* = 0.98).

## Discussion

### Summary

This study shows that there is relatively high support for health care providers addressing patients’ alcohol consumption in the general population. The majority of survey participants agreed completely or to a large extent that health care providers should ask about patients’ alcohol consumption on a routine basis.

However, there were significant socio-demographic differences in the extent to which participants could be categorized as holding “pro-routine” or “pro-personal” views on being asked about their drinking. Participants from the highest social grade (AB) were most likely to express a “pro-routine” attitude towards the incorporation of alcohol-related conversations in health care, whereas those from lower social grades (E,D, and C2) were significantly more inclined to express “pro-personal” views. We also found that those participants in the age categories 35–44 or 65 years or older were more likely to identify with “pro-personal” attitudes and beliefs compared with participants aged 16–24 years.

In contrast, there were few differences in attitudes towards addressing alcohol consumption in health care between drinking categories, a finding confirmed by the additional sensitivity analyses we conducted. The only item where opinions varied significantly concerned participants’ views on whether people answer honestly when health care providers ask about their drinking, with risky drinkers less likely than lower-risk drinkers to agree this was the case. Those categorised as risky drinkers were also significantly more likely to be male and aged between 16 and 24. There was a higher percentage of risky drinkers in social grade C1, but this difference was non-significant after adjusting for multiple comparisons.

### Strengths and limitations

This study benefits from the use of a structured survey to assess beliefs and attitudes about addressing alcohol consumption in health care conducted on a large representative sample of the adult population in England. To the best of our knowledge, this is the first survey-based study in England to investigate this issue. A further strength is the use of the validated AUDIT questionnaire to support our categorisation of participants into lower-risk or risky drinkers.

However, as with other population-based surveys, a number of limitations should be acknowledged. First, there is a risk that participants will underestimate or fail to report their drinking. Previous research suggests that even in the context of a confidential, online survey, social desirability bias leads some individuals to under-report the amount and under-estimate the consequences of their alcohol consumption [33]. Second, although our findings have international relevance for the alcohol prevention field, they are based on data gathered in a single country. Thus interpretation of our results should take into account the relatively unique national health care context in England, where services are free to all at the point of care. Third and finally, while the sample was designed to be representative, there is a risk of bias in terms of the characteristics of those who agree to participate, alongside increased likelihood that certain social groups (students, the homeless and other vulnerable populations) will be under-represented [34].

### Comparison with other literature

Our findings are broadly consistent with those from previous studies conducted in clinical populations which indicate that patients are generally favourable to discussing their drinking with health care providers [27, 28, 35–38]. This contrasts with a key theme from implementation research exploring the provider perspective on such conversations, which suggests health care providers find alcohol a difficult and sensitive topic to raise in consultations, and are worried about offending their patients in doing so [18, 39, 40]. On balance, our results imply such concerns are relatively unfounded. We did however detect a significant difference in attitudes by social grade, with participants from lower social grades less likely than those from the highest social grade (AB) to support health care providers routinely addressing patients’ alcohol consumption. Other research has found that some primary care providers are actually more likely to ask patients from lower socio-economic groups about their drinking [41], with social-distancing suggested as potentially influencing this behaviour, meaning that general practitioners (GPs) find it easier to ask people they perceive less like themselves about alcohol [42].

There is substantial evidence that alcohol-related morbidity and mortality is more commonly experienced by those in lower socio-economic groups, despite the fact that overall they report drinking the same or less than those from higher socio-economic groups: a phenomenon known as the ‘alcohol harm paradox’ [43]. Recent research based on the Health Survey for England found that although lower socio-economic status was associated with lower likelihoods of exceeding recommended alcohol consumption, there was a higher likelihood of exceeding the more extreme drinking thresholds in this group [44]. Results from previous Alcohol Toolkit Studies have also highlighted that high-risk drinking spans the spectrum of socio-demographic groups [45]. Our study detected higher levels of risky drinkers in lower socio-economic groups but this difference was not significant.

Interestingly, drinking status (whether participants were low or risky drinkers) was not associated with attitudes towards being asked about alcohol in health care. This contrasts with Nilsen et al’s 2011 Swedish population survey, which found lower levels of support for routinely asking patients about their alcohol consumption amongst excessive and hazardous drinkers [29]. Our results also differed from the Swedish study in terms of the association between the age of respondents and their alcohol-related attitudes and beliefs. In Nilsen et al, younger participants were less positive about being routinely questioned about their drinking by health care providers [29]. In this study, the opposite was the case, with younger participants (aged 16–24 years) significantly less likely to view such conversations as a personal matter (“pro-personal”) compared to older respondents. This finding echoes other research suggesting that older people may be reluctant to disclose details of their drinking and lacking in knowledge of the effects of alcohol intake on different aspects of their health [46]. Moreover, there is evidence from previous studies conducted in primary care in England and elsewhere that despite awareness of the specific and complex risks that excessive alcohol consumption present to this population, providers themselves can be reticent about asking older adults about their drinking [39, 47].

### Implications for policy and practice

These findings have several implications for policy-makers and health care providers. Despite substantial evidence for the effectiveness of screening and brief interventions [3], a well-established theme in the alcohol intervention implementation literature concerns health care providers’ anxieties about asking patients about their drinking. Our headline finding that the general population in England are mostly supportive of health care providers routinely asking about alcohol should help allay such concerns. This message also suggests that the public are broadly supportive of the direction of travel in English alcohol prevention policy in recent years, where primary care providers in particular are encouraged to regularly ask their patients about alcohol consumption and other lifestyle behaviours. If anything, given current national policy targets newly registered patients and specific patient groups only for routine alcohol screening, it appears it would be acceptable to ramp up provision to a more universal approach.

At the same time, our results highlight that certain population groups (older adults and those in lower socio-economic groups) may be less positively inclined towards routine delivery of alcohol prevention activities in health care settings. Clinicians and other health care workers should be mindful that for such individuals at least, alcohol may remain a ‘difficult business’ [18], with a more sensitive and tailored approach required. Further, whilst public attitudes may be largely positive towards the routine delivery of alcohol interventions in primary care, other research confirms the need for ongoing training and support provision to ensure health care providers possess the necessary knowledge, self-efficacy, skills and awareness to confidently discuss drinking with their patients [48, 49]. Finally, and importantly, whilst measures to address the concerns of providers and recipients of preventative alcohol activities are of course important, successful implementation demands we improve the underlying structures and support systems that shape their delivery. Thus, future policy programmes should draw on the lessons learned from Phase IV of the WHO study [50] and the US-based SAMHSA SBIRT initiative [51], and include measures to improve the wider social and political context alongside those focussed at either individual health care providers or patients.

## Conclusion

In conclusion, this study finds that most people agree that health care providers should routinely ask about patients’ alcohol consumption. However, older adults and those in lower socio-economic groups are less supportive. Drinking status appears unrelated to whether people believe that alcohol is a personal matter and not something health care providers should ask about.

## Abbreviations

ATS: Alcohol Toolkit Study
AUDIT: Alcohol Use Disorders Identification Test
NHS: National Health Service
NRS: National Readership Survey
